# Retraction Note: Loss of pigment epithelium-derived factor: a novel mechanism for the development of endocrine resistance in breast cancer

**DOI:** 10.1186/s13058-023-01711-7

**Published:** 2023-09-19

**Authors:** Rifat Jan, Min Huang, Joan Lewis-Wambi

**Affiliations:** 1 Cancer Biology Program, The Research Institute of Fox Chase Cancer Center, 333 Cottman Avenue, Philadelphia, PA 19111 USA; 2 Department of Pathology, The Research Institute of Fox Chase Cancer Center, 333 Cottman Avenue, Philadelphia, PA 19111 USA

**Retraction Note to: Breast Cancer Research 2012, 14:R146** https://doi.org/10.1186/bcr3356

The Editor-in-Chief has retracted this article at the Corresponding Author's request. After publication, concerns were raised regarding the western blot images presented in the figures. Specifically:

- Figure 1a beta-actin bands appear highly similar to Fig. 5b in the corresponding author's earlier article [1].
  Figure 7d pSer167-ER-alpha blot appears highly similar to the MCF:2A BCL-2 blot in Fig. 4a in the authors' earlier article [2].
- Figure 2b pER-Ser118 blot appears to contain an “invisible” band in lane 3.
- Figure 3b PEDF blot appears to have a vertical straight line break in the backgrounds between lanes 2 and 3.
- Figure 4a PEDF lanes 2 and 3 appear highly similar to p70S6K lanes 1 and 2, respectively.
- Figure 7d pAKT blot appears highly similar to Fig. 4d mitochondria Cyt c in [2].
  Figure 6d PBS and rPEDF images appear highly similar to Fig. 6d BSO and E2, respectively, in [2].

The Corresponding Author has stated that some of the blots from their other projects were mistakenly used in this article. The Editor-in-Chief therefore no longer has confidence in the presented data.

The authors have been offered to submit a revised manuscript with the correct data for further peer review.

Min Huang and Joan Lewis-Wambi agree to this retraction. The publisher has not been able to obtain a current email address for Rifat Jan.
